# Incidence and Risk Factors of Post‐Lumbar Puncture Headache in Patients With Cognitive Impairment

**DOI:** 10.1002/brb3.70597

**Published:** 2025-05-30

**Authors:** Xinhui Wang, Yuchen Qiao, Yuchen Liang, Jinming Han, Hongyan Duan, Yi Tang, Qi Qin

**Affiliations:** ^1^ Department of Geriatrics Henan Provincial People's Hospital, People's Hospital of Zhengzhou University Zhengzhou China; ^2^ Innovation Center for Neurological Disorders, Department of Neurology Xuanwu Hospital, Capital Medical University, National Center for Neurological Disorders Beijing China; ^3^ Capital Medical University Beijing China; ^4^ Department of Neurology, Xuanwu Hospital Capital Medical University Beijing China

**Keywords:** assessment tools, cognitive impairment, lumbar puncture, post‐lumbar puncture headache (PLPH)

## Abstract

**Background:**

Cognitive impairment is prevalent among the elderly population. Cerebrospinal fluid (CSF) biomarker tests are employed to facilitate timely and differential diagnosis of cognitive dysfunction.

**Objective:**

To investigate incidence and risk factors of post‐lumbar puncture headache (PLPH) in Chinese geriatric patients with cognitive impairment.

**Methods:**

A total of 254 consecutive patients with cognitive impairment were recruited between March and September 2023 and categorized into two groups: mild cognitive impairment (MCI) and dementia. Different scales, such as the short‐form of the McGill pain questionnaire (SF‐MPQ), facial visual analogue scale (F‐VAS), revised Wong‐Baker Assessment of Facial Expression Pain (FPS‐R), and Chinese version of Pain Assessment Scale for Advanced Dementia (C‐PAINAD), were used to evaluate the incidence of headache after lumbar puncture. Univariate and multivariate factor analyses were conducted to identify potential risk factors, with the most influential predictors included in regression models.

**Results:**

Among patients with cognitive disorders, the incidence of PLPH was 24.8%. The incidence and duration of headache did not differ significantly between the MCI and dementia groups. The average time of post‐lumbar puncture headache (PLPH) was 22.9 h. There was no significant difference in headache scale scores between MCI patients. The incidence of PLPH in women with cognitive impairment was higher than that in men. The incidence of PLPH in cognitive impairment patients with a history of headache was significantly higher than that in cognitive impairment patients without a history of headache. However, no significant differences were observed in years of education, number of lumbar punctures, length of bed rest, and oral rehydration volume.

**Conclusion:**

PLPH is more commonly observed in women, individuals with a history of headache, younger age, or lower body mass index (BMI). C‐PAINAD and SF‐MPQ are the preferred assessment tools for evaluating PLPH in patients with cognitive impairment.

## Graphical abstract

This cohort study of 254 Chinese elderly patients with cognitive impairment revealed female gender, headache history, younger age, and lower BMI as key risk factors for post‐lumbar puncture headache (PLPH), with C‐PAINAD and SF‐MPQ being optimal assessment tools.

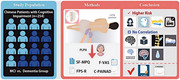

## 1 Introduction

Due to the significant rise in the elderly population throughout the world, cognitive impairment has emerged as a prevalent worldwide health concern (Gong et al. 2022). Cognitive impairment can be divided into mild cognitive impairment (MCI) and dementia according to disease severity (Albert et al. 2011). MCI represents a transitional stage between healthy aging and dementia (Anderson 2019), and the risk of MCI developing into dementia can be significantly increased. The incorporation of biomarkers into Alzheimer's disease (AD) dementia criteria in the NIA‐AA dementia diagnostic guidelines in 2011 and 2018 (Albert et al. 2011; Jack et al. 2018) recommended cerebrospinal fluid (CSF) testing as a routine examination for dementia. Lumbar puncture is required to obtain CSF, with the most common complication being post‐lumbar puncture headache (PLPH) (Headache Classification Committee of the International Headache Society 2013) being a common complication resulting from decreased intracranial pressure due to CSF leakage (Ahmed et al. 2006). Various factors can impact the occurrence of PLPH, especially in individuals with cognitive impairment who may experience language disruptions. Consequently, it is essential to focus on identifying risk factors associated with PLPH in individuals with cognitive impairment.

This study investigates PLPH incidence and risk factors in cognitively impaired patients through a multidimensional assessment framework addressing critical gaps in existing research. While prior studies report variable PLPH rates (2%–11.9%) (Blennow et al. 1993; Hindley et al. 1995; Moulder et al. 2017; Soo et al. 2022) in this population, they predominantly examined non‐modifiable technical factors like needle gauge (Peskind et al. 2005) and CSF volume (Cognat et al. 2021), neglecting clinically actionable variables such as preexisting headache history, post‐procedural recumbency duration, and fluid replacement volume‐factors. Furthermore, traditional reliance on unimodal pain scales like Numerical Rating Scales (NRS) proves inadequate for patients with communication deficits, as it fails to capture the complex pain manifestations in this population. Our protocol overcomes these limitations by implementing a multimodal assessment battery combining SF‐MPQ, F‐VAS, FPS‐R, and C‐PAINAD, enabling precise differentiation between procedure‐related headaches and preexisting pain syndromes. SF‐MPQ has demonstrated validity in Chinese populations (Wang et al. 2017). F‐VAS shows cross‐cultural reliability but requires literacy adaptation (Albert et al. 2011). FPS‐R exhibits comparable psychometrics globally (Hicks et al. 2001). The C‐PAINAD scale was specifically validated for Chinese dementia patients (Lin et al. 2010), addressing cultural communication barriers in cognitive impairment cohorts.

Beyond pain characterization, we systematically documented modifiable risk parameters including lumbar puncture frequency, initial/final CSF pressure measurements, CSF drainage volume, bed rest duration, and 24‐h oral rehydration intake. This dual approach not only enhances diagnostic accuracy but also identifies potentially intervenable risk factors, addressing the current clinical reliance on subjective chief complaints for PLPH evaluation in cognitive impairment cohorts. By integrating advanced pain metrics with comprehensive physiological monitoring, our methodology establishes a novel paradigm for understanding PLPH pathophysiology in vulnerable neurological populations.

## 2 Materials and Methods

### 2.1 Study Population

A cohort of 254 consecutive patients diagnosed with cognitive impairment were enrolled in this research study, conducted at the Department of Geriatrics, Henan Provincial People's Hospital and Department of Neurology, Xuanwu Hospital, Capital Medical University, spanning from March 2023 to September 2023.

Inclusion criteria:

1. Patients meeting the core clinical criteria of either MCI or dementia were included.
  - Dementia: This was defined as impairment interfering with the ability to function at work or in usual activities (McKhann et al. 2011). Cognitive function was assessed using either the Mini‐Mental State Examination (MMSE) with a score of ≤ 24 (H. Li et al. 2016) or the Montreal Cognitive Assessment (MoCA) with a score of ≤ 25 (Lu et al. 2011), along with a Clinical Dementia Rating (CDR) score of ≥ 1 (Morris 1993; Huang et al. 2021). Cognitive or behavioral impairment involved at least two domains, including the ability to acquire and remember new information, reasoning and handling complex tasks, judgment, visuospatial abilities, language functions, and changes in personality, behavior, or comportment. Patients with delirium or serious mental disorders were excluded.
  - MCI: Patients with MCI did not exhibit significant interference in the ability to function at work or in usual daily activities. Their CDR score was 0.5 (Morris 1993; Huang et al. 2021). Objective evidence of impairment was observed in one or more cognitive domains, typically the memory domain.
2. Patients requiring lumbar puncture for CSF for clinical disease diagnosis were included.
3. Informed consent was obtained from all participants or their families.

Exclusion criteria:

1. Patients with contraindications to lumbar puncture were excluded, including those with elevated intracranial pressure, posterior cranial fossa space‐occupying lesion, puncture site infection, coagulation dysfunction, and spinal stenosis.
2. Patients or family members who refused lumbar puncture or had difficulty cooperating with the questionnaire survey were also excluded.

This cross‐sectional study was approved by the Ethics Committee of Henan Provincial People's Hospital (batch number: [2022] Review No. 15) and Xuanwu Hospital, Capital Medical University (batch number: Clinical Research Review [2022] 201). Written informed consent was obtained from the patients and the study was in accordance with the ethical requirements of the Helsinki Declaration.

### 2.2 Lumbar Puncture

Prior to lumbar puncture, experienced neurologists conducted neurological examinations and brain magnetic resonance imaging (MRI) on all patients to exclude mass lesions with elevated intracranial pressure. The lumbar punctures were performed with the patient lying on their side on the examination table. A lumbar puncture needle with an outer diameter of 0.9 mm was utilized, inserted from the L3‐4 intervertebral space (Engelborghs et al. 2017).

### 2.3 Cognitive Screening and Pain Assessment

We utilized MMSE, MoCA, CDR, and BADL for cognitive screening. Headache evaluation was performed at 0, 6, 24, 48, and 72 h post‐lumbar puncture using SF‐MPQ, F‐VAS, FPS‐R, and C‐PAINAD.

MMSE focuses on basic cognitive functions such as temporal orientation, immediate memory, and calculation, making it suitable for assessing moderate to severe cognitive impairment (Tannous et al. 2021). The MoCA incorporates advanced cognitive domains like visuospatial/executive functions (e.g., Clock Drawing Test) and abstract thinking, specifically designed for evaluating MCI, while being significantly influenced by educational attainment (Rookes et al. 2023). The CDR spans six domains including memory, orientation, judgment, and social affairs, simultaneously evaluating cognitive function and activities of daily living (ADLs), enabling clinical staging (CDR = 0.5 indicates suspected dementia, ≥ 1 confirms dementia diagnosis) (Vos et al. 2013). By integrating these three cognitive screening scales, we can comprehensively assess cognitive function.

SF‐MPQ includes 11 pain intensity evaluations and 4 pain emotion items, with the addition of a unidimensional 100 mm VAS for overall pain intensity assessment (Melzack 1987). The completion time is reduced to 2–5 min while maintaining the sensitivity and reliability of the original MPQ. The F‐VAS incorporates a selection of six cartoons depicting emotions ranging from happiness to neutral and pain, enhancing the intuitive and figurative nature of the scoring system (Price et al. 1983). FPS‐R requires patients to rate overall pain severity from 0 (no pain) to 10 (very much pain), accompanied by six cartoon pictures representing different facial expressions to visually express the degree of pain (Hicks et al. 2001). FPS‐R is particularly suitable for elderly patients with limited education and cognitive impairment compared to the linear VAS. It is now recognized as the primary choice for pain assessment in geriatric patients (Thong et al. 2018). The Pain Assessment in Advanced Dementia Scale (PAINAD) is a tool designed for assessing pain in elderly patients with cognitive impairment who are unable to communicate verbally. C‐PAINAD is the Chinese version of PAINAD, which has been preliminarily validated for reliability and validity (Monserrate et al. 2015). A combination of the Discomfort Scale and the Face, Legs, Activity, Crying, Consolability pain assessment tool includes five pain‐related behavioral items, each rated from 0 to 2, with a maximum score of 10 indicating pain (Lin et al. 2010). It requires assessors to observe patients for 5 min and conduct non‐verbal pain scoring, including five items: breathing, vocalization, facial expression, body language, and comforting (Hadjistavropoulos et al. 2014).

### 2.4 Data Collection

We collected demographic patient information, including gender, age, education level, headache history, height, and weight. In addition, we documented the number of lumbar punctures conducted, initial and final CSF pressure readings, the volume of fluid discharged, the duration of bed rest following the lumbar puncture, and the quantity of oral rehydration administered within 24 h after the procedure.

### 2.5 Statistical Methods

Data analysis was conducted using SPSS version 23.0 software. Descriptive statistics were used to describe ratios or component ratios of count data. The *χ^2^* test was employed for categorical variables, while non‐normally distributed data were presented as median and interquartile range. Normality was tested using the Shapiro–Wilk test. The Mann–Whitney *U* test was used to evaluate the differences in relevant variables between the two groups. To analyze influencing factors, PLPH served as the dependent variable, with statistically significant comparison indices between groups serving as independent variables. We employed logistic two‐factor regression analysis, considering *p* < 0.05 as statistically significant.

## 3 Results

### 3.1 Incidence of PLPH in Patients With Cognitive Impairment

Among patients with cognitive disorders, the incidence of PLPH was 24.8% (63/254). As illustrated in Table 1, the incidence of PLPH in the MCI group was 27.8% (22/79), while it was 23.4% (41/175) in the dementia group. The incidence of headache did not significantly differ between the MCI and dementia groups (*χ^2^* = 0.57, *p* = 0.45) (Table 1). In addition, no significant difference in the volume of CSF drainage was noted (*p* = 0.849) (Table 2).

**TABLE 1:** Incidence of PLPH in patients with cognitive impairment.

Group	Number of cases	Headache group (*n* = 63)		Non‐headache group (*n* = 191)		*u*/*χ^2^*		*p*
		Cases	Proportion	Cases	Proportion			
MCI	79	22	27.8	57	72.2		0.570	0.450
Dementia	175	41	23.4	134	76.6			

**TABLE 2:** Volume of CSF drained and differential pressure of PLPH in enrolled patients.

Group	Volume of CSF drainage (mL)	*Z*	*p*
MCI	18 (15∼21)	−0.190	0.849
Dementia	18 (15∼21)		

### 3.2 Occurrence Time and Duration of PLPH in Patients With Cognitive Impairment

**FIGURE 1:**
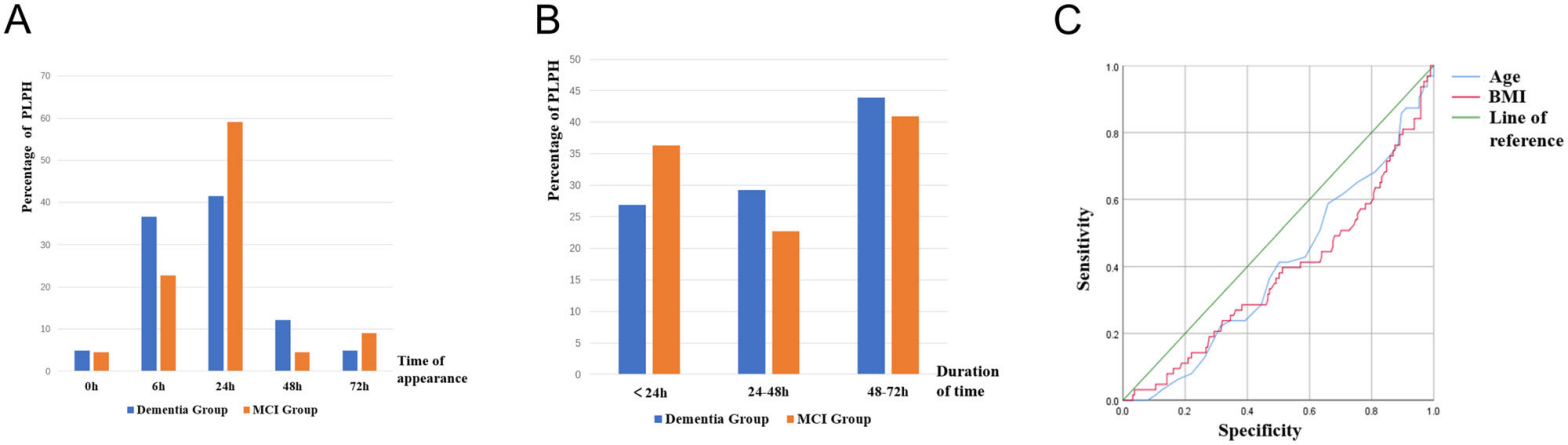
The occurrence and duration of PLPH in patients with cognitive imparment and the independent influencing factor of PLPH. (A) PLPH onset time in patients with cognitive impairment; (B) Duration of PLPH in patients with cognitive impairment, (C) A multivariate logistical regression analvsis of PLPH.

The average time for the onset of PLPH was determined to be 22.9 h. Figure 1A portrays the distribution of occurrence times, indicating no significant disparity in headache onset time between the dementia and MCI groups (χ^2^ = 3.189, *p* = 0.552) (Table 3). Figure 1B illustrates that the duration of headache was predominantly concentrated within the 48 to 72‐h timeframe. In addition, there was no statistically notable distinction in headache duration between the two groups (*χ^2^* = 0.689, *p* = 0.709), as displayed in Table 4.

**TABLE 3:** PLPH onset time in patients with cognitive impairment.

Onset time	Group		Sum (*n* = 63)	*χ^2^*	*p*
	Dementia (*n* = 41)	MCI (*n* = 22)			
0 h (%)	2 (4.88)	1 (4.55)	3 (4.76)	3.198	0.525
6 h (%)	15 (36.59)	5 (22.73)	20 (31.75)		
24 h (%)	17 (41.46)	13 (59.09)	30 (47.62)		
48 h (%)	5 (12.20)	1 (4.55)	6 (9.52)		
72 h (%)	2 (4.88)	2 (9.09)	4 (6.35)		

**TABLE 4:** Duration of PLPH in patients with cognitive impairment.

Duration	Group		Sum (*n* = 63)	*χ^2^*	*p*
	Dementia (*n* = 41)	MCI (*n* = 22)			
< 24 h (%)	11 (26.83%)	8 (36.36)	19 (30.16)	0.689	0.709
24–48 h (%)	12 (29.27%)	5 (22.73)	17 (26.98)		
48–72 h (%)	18 (43.9%)	9 (40.9)	27 (42.86)		

### 3.3 Comparison of Various Headache Assessment Scales in Patients With Cognitive Impairment

SF‐MPQ, F‐VAS, FPS‐R, and C‐PAINAD were utilized for PLPH evaluation in this study. As depicted in Table 5, no significant difference was observed in the assessment of headache scales among patients with MCI. In the dementia group, the positive rate of headache assessment followed the order SF‐MPQ > C‐PAINAD > FPS‐R > F‐VAS (from highest to lowest), with SF‐MPQ yielding the highest positive rate for headache assessment (40/175, 22.9%) and F‐VAS the lowest positive rate (25/175, 14.3%). The participation rate of pain scales in dementia patients was C‐PAINAD > SF‐MPQ > FPS‐R > F‐VAS (from highest to lowest). Among patients with MCI, there was no significant difference in the assessment of headache scales, while C‐PAINAD and SF‐MPQ scales were found to be more suitable for dementia patients.

**TABLE 5:** Comparisons of different headache assessment scales in enrolled patients.

Group	Rates	SF‐MPQ			F‐VAS	FPS‐R	PAINAD
		PRI	VAS	PPI			
MCI group (*n* = 79)	Positive rate	20, 25.3%	20, 25.3%	22, 27.8%	20, 25.3%	20, 25.3%	19, 24.1%
	Negative rate	59, 74.7%	59, 74.7%	57, 72.2%	59, 74.7%	59, 74.7%	60, 75.9%
	Participation rate	79, 100%	79, 100%	79, 100%	79, 100%	79, 100%	79, 100%
Dementia group(*n* = 175)	Positive rate	35, 20%	26, 14.9%	40, 22.9%	25, 14.3%	31, 17.7%	37, 21.1%
	Negative rate	121, 69.1%	81, 46.3%	122, 69.7%	103, 58.9%	103, 58.9%	138, 78.9%
	Participation rate	156, 89.1%	107, 61.1%	162, 92.6%	127, 72.6%	134, 76.6%	175, 100%

### 3.4 Risk Factor Analysis of PLPH in Patients With Cognitive Impairment

The analysis of risk factors for PLPH in patients with cognitive impairment revealed significant gender and previous headache history differences (*p* < 0.05, respectively) in both the headache and non‐headache groups. Among patients with cognitive impairment, the incidence of PLPH was 18.5% (23/124) in men and 30.8% (40/130) in women, with a significant difference observed in the incidence of headache between the genders (*χ^2^* = 5.082, *p* = 0.024). This suggests that PLPH occurrence in women with cognitive impairment was higher than that in men (30.8% vs. 18.5%). In addition, the incidence of PLPH was 58.3% (7/12) in individuals with a previous history of headache and 23.1% (56/242) in those without a previous history of headache, with a statistically significant difference noted between the two groups (*χ^2^* = 7.592, *p* = 0.006). This indicates that PLPH occurrence in patients with cognitive impairment with a previous history of headache was significantly higher than that in those without such a history (58.3% vs. 23.1%). However, there were no significant differences observed in years of education, the number of lumbar punctures performed, bed rest time, and oral rehydration volume within 24 h, as shown in Table 6. Notably, there were significant differences in age and BMI observed between the headache and non‐headache groups among these patients. Specifically, in patients with cognitive impairment, age was found to be associated with the occurrence of PLPH (*Z* = −2.465, *p* = 0.014), suggesting that younger individuals were more prone to suffering from PLPH. Furthermore, BMI also showed an association with the occurrence of PLPH (*Z* = −2.924, *p* = 0.003) in this patient population. However, there were no significant differences noted in CSF initial pressure, final pressure, pressure difference, and fluid drainage volume, as detailed in Table 7.

**TABLE 6:** PLPH risk factors in patients with cognitive impairment.

Information	Number of cases	Headache group (*n* = 63)		Non‐headache Group (*n* = 191)		*u*/*χ2*	*p*
		Cases	Proportion	Cases	Proportion		
Gender						5.082	0.024
Male	124	23	9.10	101	39.80		
Female	130	40	15.70	90	35.40		
Years of education						0.901	0.637
0	6	1	0.40	5	2.00		
1–6	27	5	2.00	22	8.70		
≥ 7	221	57	22.40	164	64.60		
History of headache						7.592	0.006
No	242	56	22.00	186	73.20		
Yes	12	7	2.80	5	2.00		
Numbers of lumbar punctures performed						2.251	0.325
1	169	45	17.70	124	48.80		
2	61	15	5.90	46	18.10		
≥ 3	24	3	1.20	21	8.30		
Bed rest time						2.267	0.322
< 1 h	50	9	3.50	41	16.10		
1–4 h	62	14	5.50	48	18.90		
> 4 h	142	40	15.70	102	40.20		
Oral rehydration volume (24 h)						4.071	0.131
< 1000 mL	31	9	3.50	22	8.70		
1000–2000 mL	213	49	19.30	164	64.60		
> 2000 mL	10	5	2.00	5	2.00		

**TABLE 7:** Comparison of continuous data in patients with cognitive impairment.

Information	Headache group (*n* = 63)	Non‐headache group (*n* = 191)	*Z*	*p*
Age (year)	60.00 (54.00–65.00)	63.00 (57.00–69.00)	−2.465	0.014
BMI (kg/m^2^)	22.41 (20.55–24.84)	23.66 (21.93–25.69)	−2.924	0.003
Initial pressure (mmH_2_O)	135.00 (115.00–150.00)	140.00 (120.00–170.00)	−1.703	0.089
End pressure (mmH_2_O)	70.00 (40.00–95.00)	135.00 (55.00–100.00)	−0.686	0.493
Differential pressure (mmH_2_O)	60.00 (45.00–80.00)	65.00 (50.00–95.00)	−0.727	0.467
Volume of CSF drained (mL)	18 (15.50–22.00)	18 (15.50–21.00)	−0.676	0.499

As revealed by logistic multivariate regression analysis using PLPH as the dependent variable and relevant indicators including age, gender, BMI, and history of headache as independent variables. Our findings indicated that both age and gender were significant predictors of PLPH occurrence (*p* < 0.05, respectively), as illustrated in Figure 1C and detailed in Table 8.

**TABLE 8:** Factors that influence PLPH.

Factors	*β*	Wald *χ* ^2^	SD	*p*	OR	95%CI
Age	−0.038	5.604	0.016	0.018	0.963	0.933–0.993
Gender	−0.452	2.041	0.316	0.153	1.571	0.845–2.919
BMI	−0.121	5.653	0.051	0.017	0.886	0.802–0.979
History of headache	1.039	2.633	0.640	0.105	2.826	0.806–9.906

## 4 Discussion

PLPH, the most common complication following lumbar puncture, has been reported in 9%–33% of the general population and 2%–11.9% in cognitively impaired patients in previous literature (Engelborghs et al. 2017; Cognat et al. 2021). Our study revealed a 24.8% incidence of PLPH in cognitively impaired patients through multidimensional pain assessment scales (SF‐MPQ, F‐VAS, FPS‐R, and C‐PAINAD), which exceeds previously reported rates in comparable studies. This discrepancy suggests that the pain evaluation scales employed in our current research demonstrate higher sensitivity for detecting PLPH in cognitively impaired populations.

For individuals with normal cognitive function, especially young females (particularly those under 40 years old), and those with a history of headaches, the occurrence of PLPH is more prevalent according to existing literature (Duits et al. 2016; Nielsen and Vamosi 2020). Factors such as fear of complications and the size of puncture needles may also influence the likelihood of experiencing PLPH (Armon and Evans 2005; Amorim et al. 2012). In our investigation, we identified independent risk factors for PLPH among patients with cognitive impairment, which include being female, having a previous history of headaches, and possessing a low BMI. Notably, disease severity of cognitive impairment, education level, number of lumbar punctures performed, duration of bed rest following lumbar puncture, CSF pressure, and volume of CSF drainage did not exhibit significant associations with the occurrence of PLPH.

While previous studies (Hindley et al. 1995) suggested higher PLPH incidence in MCI/normal populations compared to severely impaired individuals, their reliance on telephone‐reported headache symptoms (a method vulnerable to recall bias in cognitively impaired patients) likely compromised accuracy. In contrast, our study innovatively employed multidimensional pain assessment tools (SF‐MPQ, F‐VAS, FPS‐R, and C‐PAINAD) for PLPH evaluation, achieving a significantly improved detection rate (24.8% vs. literature‐reported 2%–11.9%), indicating superior sensitivity of these scales for cognitively impaired populations. Notably, though multiple puncture attempts (> 4) are established PLPH risk factors in general populations (Duits et al. 2016), our limited data on ≥ 3 attempts precluded definitive conclusions in this cohort. Our findings align with existing evidence showing no PLPH rate difference between immediate mobilization and 4–6 h bed rest post‐LP (Handler et al. 1982; Vilming et al. 1988; Spriggs et al. 1992; Afshinmajd et al. 2014; Choi and Chang 2018) Interestingly, while CSF volumes > 30 mL reportedly increase PLPH risk, our cohort's low‐volume extraction protocol (median 18 mL, IQR 15.5–21 mL) may explain the absence of CSF volume‐PLPH correlation, suggesting volume thresholds may differ in cognitively impaired patients.

It is recognized that women tend to be more sensitive to pain than men (Tolver et al. 2013). In line with this, the incidence of PLPH was higher among women compared to men (30.8% vs. 18.5%), underscoring the increased susceptibility of women to PLPH. These findings are consistent with previous studies by Wu et al. (2006) and Amorim et al. (2012). The higher estrogen levels in women may influence the tension of cerebral blood vessels, thereby exacerbating their response to low CSF pressure. In addition, individuals with a history of chronic headaches were found to be more prone to PLPH, with a significantly higher incidence observed among this group compared to those without such a history (58.3% vs. 23.1%). Engedal et al. (2015) conducted a sequential design study, revealing that patients with a BMI less than 20 kg/m^2^ had a significantly elevated risk of PLPH. This association may be attributed to the positive correlation between BMI and CSF opening pressure, suggesting a potential protective effect of higher BMI against CSF hypotension (Whiteley et al. 2007; Wakerley et al. 2020).

CSF leakage serves as the foundation for the development of PLPH. While preventive bed rest has been considered a measure to mitigate PLPH occurrence, several studies have yielded conflicting results regarding its efficacy (Arevalo‐Rodriguez et al. 2016). Our findings indicated no significant relationship between the duration of bed rest after lumbar puncture and the incidence of PLPH among patients with cognitive impairment. Moreover, oral or parenteral fluid supplementation has been a conventional approach for preventing PLPH, aiming to compensate for internal fluid loss by administering external fluid (Dieterich and Brandt 1988). However, a meta‐analysis encompassing 23 trials and involving 2477 participants suggested uncertainty regarding the efficacy of fluid supplementation in preventing PLPH. Similarly, our study revealed that fluid supplementation was ineffective in preventing PLPH.

We employed SF‐MPQ, F‐VAS, FPS‐R, and C‐PAINAD to assess PLPH in patients with cognitive impairment. The reliability and validity of the SF‐MPQ scale are statistically acceptable for the evaluation of Chinese patients with chronic pain (J. Li et al. 2013; Wang et al. 2017). The VAS stands out as the most commonly utilized tool for measuring pain intensity in a single dimension, boasting high accuracy, and sensitivity. F‐VAS is a linear VAS scale supplemented with several cartoon facial expressions (happy, neutral, painful, etc.), making the scoring more intuitive and vivid. It is suitable for pain assessment in patients with cognitive impairment (Wan et al. 2020). Several studies have confirmed that FPS‐R is suitable for pain assessment in Chinese patients (Miró et al. 2005; L. Li et al. 2009; Guo et al. 2015). C‐PAINAD is suitable for pain assessment in patients with cognitive impairment (Manz et al. 2000; Closs et al. 2004). The scale is also applicable to Chinese patients with with cognitive impairment (Peng et al. 2007). However, our study noted that 38.9% of dementia patients were unable to cooperate with VAS assessments, while 27.4% were unable to participate in F‐VAS assessments, indicating limitations in their use. The SF‐MPQ, which encompasses assessments for PRI, VAS, and PPI, emerged with a higher positive rate (22.9%) and wider applicability (92.6%) in our investigation. All patients with cognitive impairment underwent evaluation using C‐PAINAD, with a positive rate slightly lower than that of the SF‐MPQ (21.1% vs. 22.9%). Previous studies have suggested a low risk of PLPH in patients with MCI or dementia, potentially attributable to variations in pain assessment scales. Severe cognitive impairment may render some patients unable to report headaches, possibly resulting in an underestimation of PLPH incidence in prior investigations. Therefore, utilizing more suitable scales for headache assessment in patients with cognitive impairment is imperative. In our study, we utilized the SF‐MPQ, C‐PAINAD, FPS‐R, and F‐VAS scales, revealing a headache rate of 24.8% among patients with cognitive impairment. This underscores the suitability of the SF‐MPQ and C‐PAINAD scales for assessing headaches in this patient population.

Our study has several limitations that warrant consideration. First, the relatively small sample size from a single‐center cohort may limit the generalizability of findings, our study remains to be conducted in non‐Chinese patients, including the perception of pain across different races and ethnicity and their potential influence on the study results. Second, the analysis did not explore correlations between specific cognitive impairment etiologies (e.g., Alzheimer's disease vs. vascular dementia) and PLPH incidence, despite emerging evidence of variations in CSF dynamics across neurodegenerative subtypes. Future studies should incorporate neuroimaging markers (e.g., cerebral atrophy patterns and white matter hyperintensity volumes) and CSF biomarkers (e.g., Aβ42 and *p*‐tau) to assess whether Alzheimer's pathology or vascular burden modifies PLPH susceptibility. These refinements may ultimately inform personalized prevention strategies for vulnerable subgroups.

## 5 Conclusion

PLPH typically manifests around 23 h post‐lumbar puncture and persists for 48–72 h. Interestingly, there appears to be no significant correlation between the severity of cognitive impairment and the occurrence of PLPH. However, certain demographic factors such as being female, having a history of headaches, younger age, and lower BMI increase the likelihood of experiencing PLPH. Notably, factors such as initial pressure, final pressure, pressure difference, and CSF drainage volume do not seem to influence the occurrence of PLPH in patients with cognitive impairment. C‐PAINAD and SF‐MPQ are appropriate tools for assessing PLPH in patients with cognitive impairment.

## Author Contributions

**Xinhui Wang**: conceptualization, methodology, software, writing–original draft, writing–review and editing, data curation, funding acquisition. **Yuchen Qiao**: software, data curation, conceptualization, writing–original draft. **Yuchen Liang**: data curation. **Jinming Han**: writing–review and editing. **Hongyan Duan**: funding acquisition, methodology. **Yi Tang**: writing–review and editing, funding acquisition. **Qi Qin**: writing–review and editing, writing–original draft, conceptualization, funding acquisition.

## Ethics Statement

This cross‐sectional study was approved by the Ethics Committee of Henan Provincial People's Hospital (batch number: [2022] Review No.15) and Xuanwu Hospital, Capital Medical University (batch number: Clinical Research Review [2022] 201). Written informed consent was obtained from the patients and the study was in accordance with the ethical requirements of the Helsinki Declaration.

## Conflicts of Interest

The authors declare no conflicts of interest.

### Peer Review

The peer review history for this article is available at https://publons.com/publon/10.1002/brb3.70597

## Data Availability

The data that support the findings of this study are available from the corresponding author upon reasonable request.
